# The Occurrence and Risks of Selected Emerging Pollutants in Drinking Water Source Areas in Henan, China

**DOI:** 10.3390/ijerph16214109

**Published:** 2019-10-25

**Authors:** Donghai Wu, Ying Zhou, Guanghua Lu, Kai Hu, Jingjing Yao, Xinghou Shen, Lei Wei

**Affiliations:** 1 Key Laboratory of Integrated Regulation and Resource Development on Shallow Lakes of Ministry of Education, College of Environment, Hohai University, Nanjing 210098, China; wdh1018@hhu.edu.cn (D.W.); njlhzhouying@163.com (Y.Z.); hukaihit@hhu.edu.cn (K.H.); 2 Water Conservancy Project & Civil Engineering College, Tibet Agriculture & Animal Husbandry University, Linzhi 860000, China; yaojj0412@163.com; 3 Hydrology and Water Resources Bureau of Henan Province, Zhengzhou 450004, China; sxh@hnsl.gov.cn (X.S.); weileilazio@hotmail.com (L.W.)

**Keywords:** organic micropollutant, drinking water source, Henan Province, risk assessment

## Abstract

The occurrence of organic micropollutants (OMPs) in aqueous environments has potential effects on ecological safety and human health. Three kinds of OMPs (namely, pharmaceuticals, ultraviolet (UV) filters and organophosphate esters (OPEs)) in four drinking water source areas in Henan Province of China were analyzed, and their potential risks were evaluated. Among 48 target chemicals, 37 pollutants with total concentrations ranging from 403.0 to 1751.6 ng/L were detected in water, and 13 contaminants with total concentrations from 326.0 to 1465.4 ng/g (dry weight) were observed in sediment. The aqueous pollution levels in Jiangang Reservoir and Shahe Water Source Area were higher than that in Nanwan Reservoir and Baiguishan Reservoir, while the highest total amount of pollutants in sediment was found in Baiguishan Reservoir. Compared with pharmaceuticals and UV filters, OPEs presented higher concentrations in all investigated drinking water source areas. The highest observed concentration was triphenylphosphine oxide (TPPO, 865.2 ng/L) in water and tripentyl phosphate (TPeP, 1289.8 ng/g) in sediment. Moreover, the risk quotient (RQ) analysis implies that the determined aqueous contaminants exhibited high risks to algae and invertebrates, whereas moderate risk to fish was exhibited. The health risk assessment of aqueous OMPs by means of the hazard index (HI) indicates that the risks to adults and children were negligible. These observations are expected to provide useful information for the assessment of water quality in drinking water sources in Henan, China.

## 1. Introduction

In recent years, with the rapid urbanization and industrialization, organic micropollutant (OMP)-induced environmental pollution has attracted extensive attention [1,2,3]. Due to their wide use and incomplete elimination, OMPs have been frequently detected in various water. Although the concentrations of these pollutants were found at low levels (ng/L-μg/L) [4,5], their presence is considered to pose a threat to aquatic organisms and human health. For instance, some pharmaceuticals could increase drug resistance, making drugs ineffective and induce endocrine disruptive conditions [6,7]. Ultraviolet (UV) filters have a tendency to be accumulated in organisms [8,9], causing endocrine disruption, such as antiestrogenic and antiandrogenic activity [10,11]. Furthermore, some UV filters could inhibit the growth and immobilization of aquatic organisms [8]. It is reported that organophosphate esters (OPEs) could influence physiological activities, including the inhibition of paraoxonase and cholinesterase, nitrosative stress, and pancreatitis [12]. Unfortunately, OMP pollution has occurred in drinking water source areas, which are important resources for human beings.

According to a previous report, over 50 antibiotics were found in the Danjiangkou Reservoir in China, with the highest concentration of 149 ng/L for sulfamethoxazole (SMX) in water, and 12.1 ng/g for oxytetracycline (OTC) in sediment, respectively [13]. Pompei et al. noted that six target pharmaceutical and personal care products (PPCPs) could be identified in a Brazilian water reservoir in the range of μg/L, especially metyl 4-hydroxybenzoate (MEP) and 2-hydroxy-4-methoxybenzophenone (BP3) were detected with a frequency of 100% [14]. The total concentration of OPEs were detected with a range of 166–1530 ng/L in water and 2.82–47.5 ng/g (dry weight) in sediment of Taihu Lake, China [15]. Due to the transfer and transformation of contaminants, the OMPs in sediment may have a potential influence on water quality [16]. The occurrence of OMPs is bound to present a risk to aqueous organisms and human beings.

Therefore, the method of quantitative risk is vital for monitoring the OMP pollution levels, dealing with risk management and making regulations for drinking water source areas [17]. In general, quantitative risk assessment includes ecological risk assessment and human health risk assessment. The risk quotient (RQ) methodology, which is calculated using the ratio of the measured environmental concentration (MEC) to the predict no effect concentration (PNEC) of the target pollutants, has extensively been applied to evaluate the ecological risk assessment of OMPs [18]. The hazard index (HI) method is aimed to quantify the non-cancer risk of contaminants to human health on exposure to polluted water—the value of which is obtained from chronic daily intakes (CDIs) divided by the reference dose (RfD) [19].

Henan is a province in China that has a large population. However, the OMP pollution in drinking water sources of Henan is largely unknown, and few systematic studies have evaluated its quantitative risk. The main objectives of this work were the determination of OMP pollution and the assessment of their risks in drinking water sources of Henan, China. The concentrations of 48 OMPs that were classified into three groups (i.e., pharmaceuticals, UV filters and OPEs) were investigated, and their risks to three trophic levels and humans were assessed.

## 2. Materials and Methods

### 2.1. Chemicals and Reagents

A total of 48 chemicals were selected as target contaminants, including 35 pharmaceuticals, 8 UV filters and 5 OPEs. Detailed properties of these chemicals are provided in Table S1. The standards of chemicals were purchased from Ehrenstorfer (Augsburg, Germany) or Sigma-Aldrich (Flanders, NJ, USA), and all solvents of high-performance liquid chromatography (HPLC) grade were obtained from Merck (Darmstadt, Germany).

### 2.2. Sample Collection

**Figure 1:**
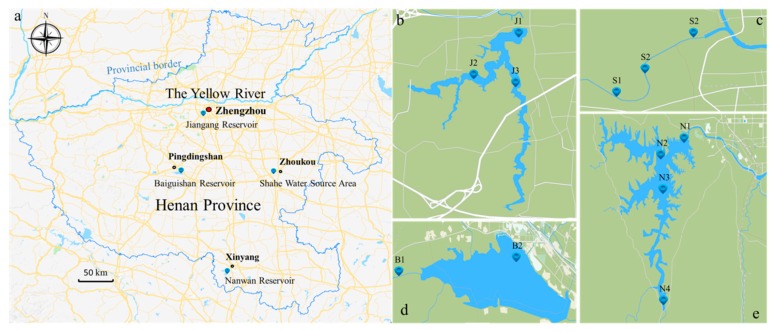
(**a**) Location of sampling area and sites, with four water source areas: (**b**) Jiangang Reservoir, (**c**) Shahe Water Source Area (**d**) Baiguishan Reservoir, and (**e**) Nanwan Reservoir.

The sample collection was conducted in Baiguishan Reservoir (B), Nanwan Reservoir (N), Jiangang Reservoir (J) and Shahe Water Source Area (S) in November 2017. The position of four drinking water source areas and sampling sites are presented in Figure 1, and detailed information is summarized in Table S2and Text S1 . Water samples were collected at 0.5 and 2.5 m below the surface and were cooled in prepared polypropylene bottles immediately during transportation to the laboratory for further treatment within 24 h. Similarly, surface sediment samples (0–10 cm) were collected with a stainless steel grab sampler and were subsequently lyophilized and stored in a freezer (−80 °C) in the laboratory for further treatment.

### 2.3. Sample Treatment and Analysis

Firstly, water samples were filtered through 0.45 μM glass fiber membranes to remove suspended particles for extraction. The procedures of the extraction method referred to previous studies [20,21,22]. In brief, 500 mL of the water sample was filtered and passed through the activated solid-phase extraction column (SPE). Then, the extracts were concentrated using a gentle stream of nitrogen and then diluted for quantitative analysis. Then, 0.5 g of sediment sample was ground, sieved, and extracted using an accelerated solvent extractor (Dionex ASE 350, Thermo Fisher Scientific, MA, USA). Afterwards, the extracts were concentrated to dryness, and then redissolved for instrumental detection. The detailed procedure was summarized in Text S2.

The instrumental analysis of target chemicals was carried out employing a Water Acquity ultra performance liquid chromatography–tandem mass spectrometry system (UPLC-MS/MS; Waters, VA, USA) with an electrospray ionization source. Extract analysis was performed with an Acquity UPLC BEH-C18 column (100 mm × 1.7 μm × 2.1 mm; Waters, VA, USA) at 40 °C. The analytic conditions are shown in Text S3 and Table S3.

### 2.4. Quality Assurance and Quality Control (QA/QC)

In order to monitor background pollution, procedural blanks, solvent blanks and field blanks were employed for each batch of 12 samples. The target pollutants were not detected in the blank samples. The limit of detection (LOD) for each chemical is defined as 3-fold of the signal-to-noise ratio (s/n) at low spiked concentrations, and the limit of quantification (LOQ) is 10-fold of the s/n. The specific LODs and LOQs were summarized in Table S4. The recoveries of pharmaceuticals and UV filters are from 72.1%~106.3% in water and 67.2%~122.1% in sediments. The recoveries of OPEs are in the range of 49.3%~68.7% in water and 76.3%~90.8% in sediments.

### 2.5. Risk Assessment

#### 2.5.1. Ecological Risk Assessment

The ecological risk of detected OMPs to algae, invertebrates and fish can be evaluated by the RQ approach, which is among the most widely used methods for risk assessment. RQs are calculated according to Equation (1) [18]:

(1) $$\mathrm{RQ}\;=\;\frac{\mathrm{MEC}}{\mathrm{PNEC}}$$

where MEC is the measured environmental concentration (ng/L) and PNEC is the predicted no-effect concentration (ng/L).

Based on the guideline of the European Commission (EC) (2003), PNEC is calculated by dividing the values of the acute toxicity or chronic toxicity for non-targeting organisms by an assessment factor (AF), where the acute toxicity refers to the median lethal concentration (LC_50_) or the mean effective concentration (EC_50_) and the chronic toxicity represents the no observable effect concentration (NOEC) [23]. The values of AF are set as 1000 for LC_50_ or EC_50_, and 100, 50 or 10 for the NOEC when the data of one, two or three trophic levels is available [24]. Generally, the risk level is classified into three grades: low risk with RQ ≤ 0.1, medium risk with 0.1 < RQ < 1, and high risk with RQ ≥ 1 [25]. The toxicity data used in this study (shown in Table S5) were collected from the literature or estimated with Ecological Structure Activity Relationships (ECOSAR).

#### 2.5.2. Health Risk Assessment

The non-carcinogenic risks from water ingestion were determined by the HI method, using Equation (2) [19,26]:

(2) $${\mathrm{HI}}_{\mathrm{ingestion}\;}=\;\frac{{\mathrm{CDI}}_{\mathrm{ingestion}}}{\mathrm{RfD}}$$

where CDI_ingestion_ is the chronic daily intake of the OMPs through ingestion (mg/(kg·day)), and RfD is the reference dose for the OMPs (mg/(kg·day)). If the values of HI > 1, this means possible adverse health effects; if HI < 1, this indicates insignificant effects [27].

CDI_ingestion_ can be estimated according to Equation (3) [28]:

(3) $$\mathrm{CDI}\;=\;\frac{C\times \mathrm{IR}\times \mathrm{EF}\times \mathrm{ED}}{\mathrm{BW}\times \mathrm{AT}}$$

where C means the concentration of OMPs in water, mg/L; IR means the water ingestion rate, 1.41 L/day for an adult and 0.87 L/day for a child; EF means the exposure frequency, 365 days/year; ED means the exposure period, 70 years for an adult and 6 years for a child; BW means the body weight, 70 kg for an adult and 20 kg for a child; and AT means the average lifespan, 25,550 days for an adult and 2190 days for a child [28].

RfD can be calculated according to Equation (4) [23]:

(4) $${\mathrm{RfD}\;=\;\mathrm{LD}}_{50}{\times 4\times 10}^{-5}$$

where LD_50_ is the acute median lethal dose of OMPs summarized in Table S5, obtained from (https://toxnet.nlm.nih.gov/).

## 3. Results and Discussion

### 3.1. The Occurrence of OMPs in Water

**Figure 2:**
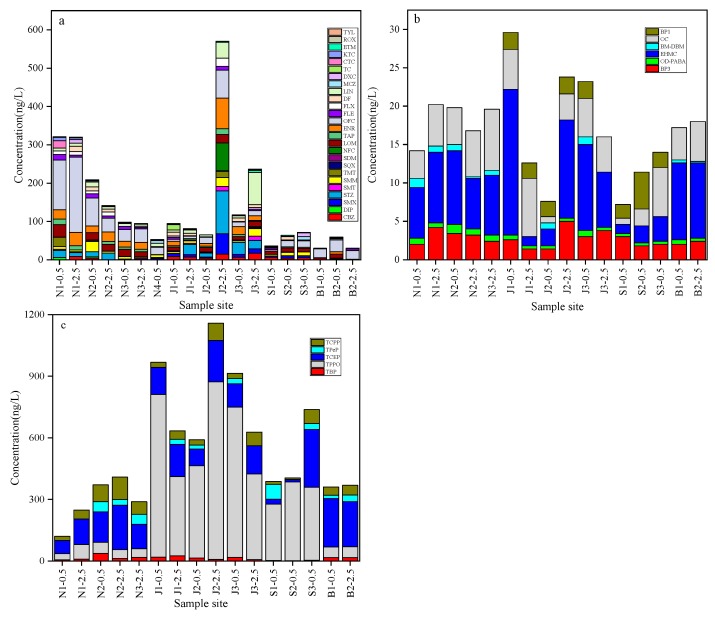
Concentrations of target pollutants in the water of four water source areas in Henan: (**a**) pharmaceuticals, (**b**) UV filters, and (**c**) OPEs (−0.5/2.5 means that the sample was collected from 0.5/2.5 m below the water surface.)

The concentrations of detected contaminants are summarized in Table S6, and the comparation of various pollutants was illustrated in Figure 2. Among 48 kinds of OMPs examined in this study, 26 pharmaceuticals, six UV filters and five OPEs were observed above the LOQ values at least once in the water. Based on the detected pollutant concentrations, the calculated total amount of OMPs ranges from 403.0 ng/L (at N3-2.5) to 1751.6 ng/L (at J2-2.5) (Figure S1). OPEs presented the highest pollution level among three sorts of OMPs, accounting for excesses of 50% of the determined total pollutant amount at most sample sites. The aqueous pollution levels of the Jiangang Reservoir and Shahe Water Source Area were higher than that of the other two regions, revealing that the pollution levels had a spatial difference. This finding appears to be related to the different consumption customs of related OMPs in these regions. Also, the aquatic environmental conditions could affect the aqueous OMP concentrations. It seems that the water depth variation had an influence on contaminant concentrations. Since the temperature of samples at 0.5 and 2.5 m below the water surface were similar, the observed phenomenon may be explained by the difference of hydrodynamics and water quality between various water layers.

For pharmaceuticals, the concentrations determined at seven sample sites were over 100 ng/L, especially at N1-0.5, N1-2.5, N2-0.5, N2-2.5, J2-2.5, J3-0.5 and J3-2.5 (Figure 2a). As commonly used antibiotics or antidepressants [29,30,31,32], the detection frequencies of sulfamonomethoxine (SMM), erythromycin (ETM), lincomycin (LIN) and fluoxetine (FLX) were over 80% in water. The detection frequency of ofloxacin (OFC) was 100%, with concentrations ranging from 3.8 ng/L (at S1-0.5) to 196.4 ng/L (at N1-2.5). OFC occupied a large proportion (>10% at most proportion) versus other pharmaceuticals, especially at B2-2.5 (~74.3%), which implies its dominant position within the pharmaceuticals detected. This may be due to the fact that OFC is one of the most widely used antibiotics and it is resistant to biodegradation [33]. Compared with the Danjiangkou Reservoir (in China) that had the highest OFC concentration of 3.6 ng/L [13], the four water source areas investigated in this work showed higher OFC pollution levels.

As shown in Figure 2b, the total concentrations of UV filters were less than 30 ng/L, ranging from 7.2 ng/L (at S1-0.5) to 29.6 ng/L (at J1-0.5). Among eight kinds of UV filters, ethylhexyl-methoxycinnamate (EHMC), octocrylene (OC), Octyl dimethyl 4-Aminobenzoic acid (OD-PABA) and BP3 were 100% detected. The dominant UV filter pollutant was EHMC, which had a mean concentration that ranged from 2.2 ng/L (in Shahe Water Source Area) to 9.9 ng/L (in Baiguishan Reservoir). This is consistent with the results in the literature, which mentions that EHMC could be frequently detected in Hongkong (93%) and Bangkok (100%) [34]. As the maximum content of BP-UV filters in personal care products [11], BP3 showed a higher detection frequency than benzophenone-1 (BP1) and benzophenone-4 (BP4) in the current study. UV filters were mainly explored for the protection of skin from UVA and UVB radiation, so they are used extensively during summer and less used in winter [5]. On the other hand, it was previously suggested that wastewater discharges and recreational activities are two main sources of UV filters in aquatic environments [11,20]. As drinking water sources, the investigated areas in this work had low wastewater and recreational pollutions, which may be reasonable for the detection of fewer UV filters than those in the reported surface water [35].

The total concentrations of OPEs ranged from 119.8 (at N1-0.5) to 1157.8 ng/L (at J2-2.5), and the mean concentrations of OPEs were 287.2, 815.5, 509.9 and 364.7 ng/L in Nanwan Reservoir, Jiangang Reservoir, Shahe Water Source Area and Baiguishan Reservoir, respectively (Figure 2c). Tributyl phosphate (TBP), triphenylphosphine oxide (TPPO), tris (2-chloroethyl) phosphate (TCEP) and tris (2-chloroisopropyl) phosphate (TCPP) were 100% detected, while the detection frequency of tripentyl phosphate (TPeP) was 62.5%. TPPO and TCEP were the dominant OPE pollutants, and the median concentrations of the former ranged from 48.0 (in Nanwan Reservoir) to 607.5 ng/L (in Jiangang Reservoir), while concentrations of 105.5 (in Shahe Water Source Area) to 227.7 ng/L (in Baiguishan Reservoir) were observed for the latter. The above OPE amounts were comparable to those reported in Taihu Lake (in China, with highest total concentration of 1530 ng/L and TCEP was the predominant OPE pollutants) [15]. Considering the examined areas are drinking water sources, OPE pollution should not be ignored.

### 3.2. The Occurrence of OMPs in Sediment

**Figure 3:**
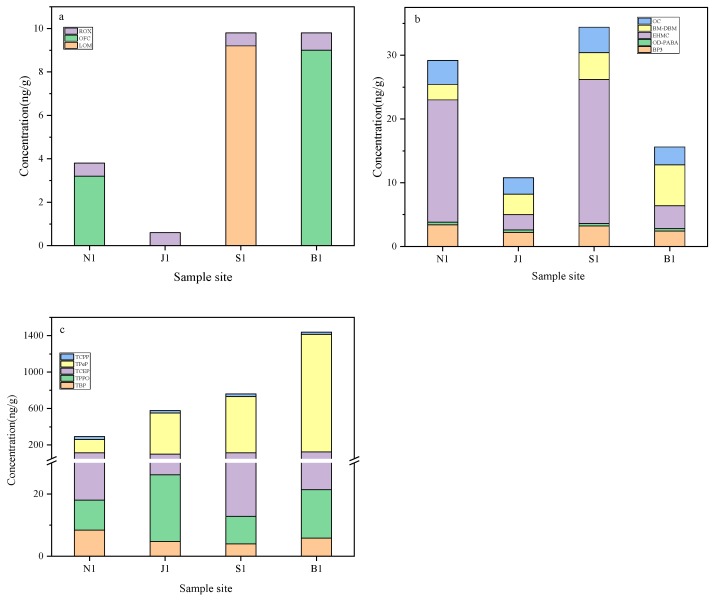
Concentrations of the target pollutants in the sediments of four water source areas in Henan: (**a**) pharmaceuticals, (**b**) UV filters, and (**c**) OPEs.

Figure 3 and Table S7 show the contents of OMPs in sediments, including three pharmaceuticals, five UV filters and five OPEs. The total concentrations of OMPs ranged from 326.0 ng/g (dry weight) in Nanwan Reservoir to 1465.4 ng/g in Baiguishan Reservoir (Figure S2), suggesting a difference in pollutant spatial distribution and a high pollution level in Baiguishan Reservoir. Similar to the OMPs in water, OPEs were the dominant contaminants, with the highest percentage accounting for the total pollutants up to 89.9%.

It was reported that pharmaceuticals had relatively low detected frequencies in sediments than in water, in Taihu Lake (China) [36]. The results in this work were similar, with the detected frequencies of pharmaceuticals in sediment much lower than those in water, which may be partly attributed to the fact that some of them have low log *K*_oc_ values (Table S1). The Shahe Water Source Area and Baiguishan Reservoir exhibited high pharmaceutical pollution levels, and lomefloxacin (LOM) and OFC were the main pharmaceutical contaminants in these two areas, respectively (Figure 3a). Moreover, five UV filters were detected in sediments (Figure 3b), especially for the lipophilic EHMC that had much higher concentrations than other UV filters in Nanwan Reservoir and Shahe Water Source Area. As shown in Figure 3c, OPEs had total concentrations from 293.0 to 1440.0 ng/g, implying that OPEs were the main pollutants among the detected OMPs in sediments. TPeP was the predominant OPE contaminant, with a concentration in the sediment of up to 1289.8 ng/g (at the B1 sampling site). The high lipophilicity (log *K_ow_* = 5.29, Table S1) of TPeP implies that it could be easily adsorbed in sediment, which is a possible reason for why it had low concentrations in water (Figure 2) and high concentrations in sediment.

### 3.3. Risk Quotients of OMPs in Water

**Figure 4:**
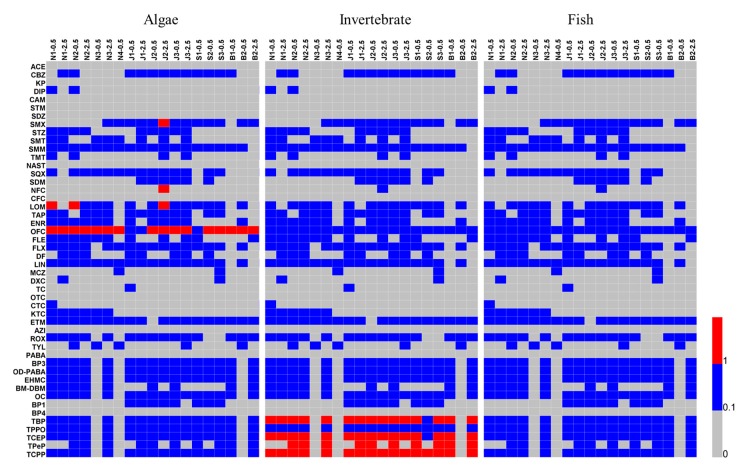
The distribution of calculated risk quotient (RQ) values of each detected compound in water.

The presence of OMPs may affect organisms in aquatic ecosystems. The ecological risk assessment is supposed to be estimated from different trophic levels, including algae, invertebrates and fish [20,34]. Based on the determinate aqueous OMPs, the corresponding ecological risks to aquatic organisms were evaluated, and the values of individual contaminants were shown in Figure 4 and Table S8. Generally, ∑OMPs showed different risk levels towards various trophic subject organisms. For algae, ∑OMPs yield the highest risk quotient, with a value of 20.81. Figure S3 illustrates that ∑Pharmaceuticals had a higher risk than other OMPs. That the abundant pharmaceuticals were detected is a possible reason for this phenomenon, and that some pharmaceuticals had high risk quotients maybe another explanation. For instance, the risk quotients of SMX, LOM and OFC were much higher than 1.0 (Figure S3), which is the value of the standard line for high risk. In particular, OFC had a value of RQ up to 19.6, comparable to the results studied in Chaohu Lake (China) [37]. In contrast, OPEs and UV filters had RQs less than 1.0. On the other hand, OPEs were the main pollutants that cause ecological risk to invertebrates, while pharmaceuticals and UV filters showed low values of RQs (Figure 4). This may be attributed to the fact that OPEs had high concentrations in water as mentioned above (Figure 2c) and the values of their PNECs are low. For fish, all of the OMPs presented a low risk, with a highest RQ value of 0.86. UV filters were the predominant contaminants due to their high toxicity to fish (Table S5). For example, OC and EHMC with PNEC < 100 ng/L are supposed to pose a threat to fish. As drinking water sources, four areas showed high ecological risks to algae and invertebrates, which demands more attention for this phenomenon.

### 3.4. Health Risk Assessment for Ingestion

The quality of the drinking water source is associated with health of residents. Hence, the non-carcinogenic human health risks posed by exposure to OMPs were assessed in the form of HIs (Table 1). It was observed that a single pollutant showed a low HI (<0.01) and the HI of ∑OMPs was excessively less than 1, indicating a low threat to people whose drinking water sources are from these areas. The HIs for adults and children were 2.01 × 10^−8^–3.16 × 10^−4^ and 4.35 × 10^−8^–6.82 × 10^−4^, respectively. The higher potential risk towards children in this work is consistent with the results in previous reports [26,28,38].

Compared with OPEs, pharmaceuticals and UV filters presented much lower health risks. As pharmaceuticals, OFC and FLX exhibited relatively high risks, with HIs of 3.92 × 10^−5^ and 9.30 × 10^−5^ for children, respectively. For UV filters, BP3 and OC were the dominant pollutants accounting for the HIs. Whereas, for OPEs, TPPO, TCEP and TCPP posed a higher health risk than the others. The predominant OMPs in potential health risk assessments corresponded to the results of the determination of occurrence and the assessment of ecological risk (Figure 2 and Figure 4). It should be noted that the investigated water samples were raw water from drinking water sources. Before intake by humans, a purification process is needed. Although evidence has shown that PPCPs could be partly removed during drinking water treatment [14,39], the risk of OPEs in purified water may be increased [40].

## 4. Conclusions

The occurrence and risk of OMPs have been reported mainly focused on rivers. However, fewer studies investigated OMPs in drinking water source areas. This work revealed that abundant OMPs occurred in the four important drinking water source areas of Henan Province, China. Pharmaceuticals had the most kinds of pollutants, while OPEs exhibited the highest concentrations both in water and sediment. Also, six UV filters were observed with trace concentrations. Generally, Jiangang Reservoir and Shahe Water Source Area had higher pollutants than the other two areas. The calculated total concentrations of aqueous OMPs ranged from 403.0 to 1751.6 ng/L, and 326.0 to 1465.4 ng/g OMPs were detected in sediments. Based on the determined OMPs, risk analyses indicated that the pharmaceuticals had high ecological risks towards algae and OPEs yield a high ecological risk to invertebrates. The human risks caused by OMPs were negligible, but OMP pollution cannot be ignored for their adverse effects on the aquatic ecological system. It should be noted that although the sampling in Henan Province in November is convenient and representative, further investigations of emerging pollutant characteristics in drinking water source areas at various times are needed to evaluate their influences more comprehensively.

## Figures and Tables

**Table 1:** The hazard index (HI) of three groups of pollutants for children and adults by ingestion.

Sample Site	∑HI_pharmaceuticals_		∑HI_UV filters_		∑HI_OPEs_	
	Adult	Child	Adult	Child	Adult	Child
N1-0.5	8.11 × 10^−5^	1.75 × 10^−4^	4.99 × 10^−7^	1.08 × 10^−6^	3.75 × 10^−5^	8.09 × 10^−5^
N1-2.5	5.96 × 10^−5^	1.29 × 10^−4^	8.30 × 10^−7^	1.79 × 10^−6^	7.79 × 10^−5^	1.68 × 10^−4^
N2-0.5	5.28 × 10^−5^	1.14 × 10^−4^	7.15 × 10^−7^	1.54 × 10^−6^	1.04 × 10^−4^	2.24 × 10^−4^
N2-2.5	3.48 × 10^−5^	7.51 × 10^−5^	8.22 × 10^−7^	1.78 × 10^−6^	1.16 × 10^−4^	2.50 × 10^−4^
N3-0.5	1.10 × 10^−5^	2.38 × 10^−5^	n.a.	n.a.	n.a.	n.a.
N3-2.5	1.54 × 10^−5^	3.34 × 10^−5^	9.69 × 10^−7^	2.09 × 10^−6^	7.61 × 10^−5^	1.64 × 10^−4^
N4-0.5	1.43 × 10^−5^	3.09 × 10^−5^	n.a.	n.a.	n.a.	n.a.
J1-0.5	3.31 × 10^−5^	7.14 × 10^−5^	8.29 × 10^−7^	1.79 × 10^−6^	3.41 × 10^−4^	7.36 × 10^−4^
J1-2.5	1.70 × 10^−5^	3.67 × 10^−5^	9.78 × 10^−7^	2.11 × 10^−6^	2.08 × 10^−4^	4.50 × 10^−4^
J2-0.5	1.33 × 10^−5^	2.88 × 10^−5^	2.93 × 10^−7^	6.33 × 10^−7^	2.01 × 10^−4^	4.34 × 10^−4^
J2-2.5	1.20 × 10^−4^	2.59 × 10^−4^	8.12 × 10^−7^	1.75 × 10^−6^	4.01 × 10^−4^	8.67 × 10^−4^
J3-0.5	2.03 × 10^−5^	4.39 × 10^−5^	8.37 × 10^−7^	1.81 × 10^−6^	3.14 × 10^−4^	6.78 × 10^−4^
J3-2.5	4.32 × 10^−5^	9.32 × 10^−5^	7.22 × 10^−7^	1.56 × 10^−6^	2.14 × 10^−4^	4.63 × 10^−4^
S1-0.5	1.37 × 10^−5^	2.96 × 10^−5^	3.90 × 10^−7^	8.42 × 10^−7^	1.14 × 10^−4^	2.45 × 10^−4^
S2-0.5	1.46 × 10^−5^	3.15 × 10^−5^	6.25 × 10^−7^	1.35 × 10^−6^	1.46 × 10^−4^	3.16 × 10^−4^
S3-0.5	2.44 × 10^−5^	5.27 × 10^−5^	8.98 × 10^−7^	1.94 × 10^−6^	2.31 × 10^−4^	4.98 × 10^−4^
B1-0.5	6.72 × 10^−5^	1.45 × 10^−5^	5.59 × 10^−7^	1.21 × 10^−6^	1.03 × 10^−4^	2.23 × 10^−4^
B2-0.5	1.26 × 10^−5^	2.71 × 10^−5^	n.a.	n.a.	n.a.	n.a.
B2-2.5	3.44 × 10^−6^	7.42 × 10^−6^	6.87 × 10^−7^	1.48 × 10^−6^	1.02 × 10^−4^	2.20 × 10^−4^

n.a.: not available.

## Supplementary Materials

The following are available online at https://www.mdpi.com/1660-4601/16/21/4109/s1, Text S1: Information on four drinking water source areas in Henan, China. Text S2: Details of sample extraction. Text S3: Details of instrumental analysis. Table S1: Physicochemical properties of the selected compounds in this study. Table S2: Basic information on sampling site and water quality parameters in different sampling sites. Table S3: Experimental conditions used for LC–MS/MS. Table S4: Recoveries, LODs and LOQs of target compounds from surface water and sediment. Table S5: Aquatic toxicity data and the calculated PNEC values (ng/L) for algae, invertebrates and fish. Table S6: The concentrations of the detected organic micropollutants in water at each sampling site. Table S7: The concentrations of the detected organic micropollutants in sediment at each sampling site. Table S8: The RQ values of the detected organic micropollutants in water at each sampling site. Figure S1: Concentrations of target pollutants in the water of four water source areas in Henan, China. Figure S2: Concentrations of target pollutants in sediment of four water source areas in Henan, China. Figure S3: RQs of target pollutants to algae (a), invertebrates (b) and fish (c) in the water of four water source areas in Henan, China.
