# Supplementary material for: The Occurrence and Risks of Selected Emerging Pollutants in Drinking Water Source Areas in Henan, China
Source: Int J Environ Res Public Health. 2019 Oct 25;16(21):4109. doi: 10.3390/ijerph16214109 (PMC6862118; doi:10.3390/ijerph16214109)
Supplement: Supplementary file 1 [file ijerph-16-04109-s001.zip › Supplementary Material/ijerph-618322-suppl proof (completed).docx]

Supplementary Materials: Occurrence and risks of selected emerging pollutants in drinking water source areas in Henan, China

Table S1. Physicochemical properties of the selected compounds in this study.

Table S2. Basic information of sampling site and water quality parameters in different sampling sites.

Table S3. Experimental conditions used for LC-MS/MS.

Table S4. Recoveries, LODs and LOQs of target compounds from surface water and sediment, with spiked concentration of 100 ng/L and 50 ng/g, respectively.

Table S5. Aquatic toxicity data and the calculated PNEC values (ng/L) for algae, invertebrate and fish.

Table S6. The concentrations of the detected organic micropollutants in water at each sampling site.

Table S7. The concentrations of the detected organic micropollutants in sediment at each sampling site.

Table S8. The RQ values of the detected organic micropollutants in water at each sampling site.

Text S1. Information of Four Drinking Water Source Areas in Henan, China

Baiguishan Reservoir, Nanwan Reservoir, Jiangang Reservoir and Shahe Water Source Area are important water source areas in Henan, China. Baiguishan Reservoir is the one of the drinking water sources of Pingdingshan City, with a water supply population of ~0.8 million and an annual water supply demand of ~68.7 million tons. Nanwan Reservoir is now the water source of centralized water supply in Xinyang City, with a water supply population of ~0.7 million and an annual water supply demand of ~117 million tons. Jiangang Reservoir has a total storage capacity of ~68.2 million m^3^, and it was designated as a standby drinking water source of Zhengzhou City in 2000. Zhoukou section of Shahe River is one of the drinking water sources of of Zhoukou City, with a water supply population of ~0.25 million and an annual water supply demand of ~20.8 million tons.

Text S2. Details of Sample Extraction

With regard to water samples extraction, different categories of chemicals demand according extraction methods. For pharmaceuticals extraction, 500 ml of filtered water samples were primarily required to undergo Oasis HLB SPE cartridges (Waters, Milford, MI, USA) at a rate of about 5 ml/min, which were preconditioned with 5 mL methanol and 5 mL ultrapure water. Next, cartridges were washed with 5 ml ultrapure water containing 5% methanol and dried at vacuum for 30 min. Then, the cartridges were subsequently eluted with 6 ml methanol and 6 ml methanol/acetone for samples extraction. Finally, extracts were evaporated to dryness under a gentle stream of nitrogen and redissolved with 1 ml methanol for analysis. Lyophilized sediment sample (0.5 g) was extracted by an accelerated solvent extractor (Dionex ASE350, CA, USA). The extracting solvent was the mixtures of acetone and methanol (1:1, v/v). The extraction was operated in three cycles at 70 °C and 1500 psi in 5 min of static extraction period. The extract was then concentrated to 1 mL using a rotary evaporator (Büchi R200, Labortechnik, Switzerland).

For UV filters pretreatment, 500 mL of water sample was also filtered and passed through an Oasis HLB SPE cartridge at a flow rate of 5 mL/min, which was preconditioned with dichloromethane (6 mL), methanol (6 mL) and ultrapure water (6 mL). And then, water sample was loaded and the cartridge was washed with 10 mL ultrapure water and dried under vacuum for 0.5 h. The cartridge was then eluted using 2 × 5 mL methanol/dichloromethane (1:1, v/v), and the extract was further evaporated to 1 mL under a gentle stream of nitrogen for further analysis. Lyophilized sediment sample (0.5 g) was also extracted by the accelerated solvent extractor with the mixtures of methanol and ethyl acetate (1:2, v/v) as the extracting solvent. The extraction was carried out in three cycles at 160 °C and 1500 psi in 5 min. The extract was also concentrated to 1 mL using the rotary evaporator.

For OPEs, the collected water samples (500 mL) need to be filtered by 0.45 μm cellulose acetate membrane, and the filtrate is extracted by SPE method. Firstly, HLB extraction column was activated by 3 mL acetone, 3 mL methanol and 3 mL ultra-pure water. Then 20 ng TBP-d27 was added to the filtered water sample. After the SPE extraction column was dried in low vacuum, the elution was carried out by 6 mL acetone. After the elution liquid nitrogen was blown to 0.1 mL, the residue was dissolved in methanol to 1 mL. Lyophilized sediment sample (0.5 g) was added into the extraction tank, and mixed with the added diatomite dispersant. TBP-d27 of 20 ng was added. The bottom and top parts of the extraction tank were respectively equipped with matching glass fiber filter membrane. In the extraction process, three five-minute cycles of static time were set. The extraction conditions were set at 100°C and 1500 psi. The extraction solvent was a mixture of dichloromethane and ethyl acetate (2:1, v:v). The extract was concentrated to less than 1 mL, and the residue was dissolved in methanol to 1 mL for further analysis.

Text S3. Details of Instrumental Analysis

For pharmaceuticals, in positive ionization mode the mobile phase consisted of eluent A (ultrapure water with 0.1% formic acid, v/v) and eluent B (acetonitrile). The gradient program was: 80% A held for 0.5 min, decreased linearly to 10% A in 5.5 min and kept for 2 min, and then increased to 80% A in 1.5 min ; In negative ionization mode, the mobile phases were ultrapure water with 0.02 mM ammonium acetate (A) and acetonitrile (B), the gradient was 80% A for 0.5 min, decreased linearly to 5% A in 5.5 min, and then increased to 80% A in 0.5 min. 5 μL mobile phases was injected for each analysis, and the flow rate was 0.3 mL/min.

For UV filters, the mobile phase were ultrapure water and methanol (98:2, v/v) containing 0.1% (v/v) formic acid (A) and methanol containing 0.1% formic acid (B). The gradient program was: 90% A for 0.25 min, decreased to 2% A in 4.75 min and kept for 1.5 min, and then increased to 90% A. A post run time of 2 min was applied before the next injection with flow rate of 0.4 mL/min.

For OPEs, the mobile phase were 0.1% formic acid and methanol. The gradient was: 90% A and 10%B for 2 min, 50% A and 10%B for 5 min, 5% A and 95% B for 1.5 min and then 90% A and 10% B for 1.5 min. 5 μL mobile phases was injected for each analysis and the flow rate was 0.4 mL/min.

**Figure S1.** Concentrations of target pollutants in water of four water source areas in Henan, China.

**Figure S2.** Concentrations of three groups of target pollutants in sediment of four water source areas in Henan.

**Figure S3.** RQs of target pollutants to algae (**a**), invertebrate (**b**) and fish (**c**) in water of four water source areas in Henan, China.

Reference

1. Yan, Zhenhua; Haohan Yang; Huike Dong; Binni Ma; Hongwei Sun; Ting Pan; Runren Jiang; Ranran Zhou; Jie Shen; Jianchao Liu; et al. "Occurrence and Ecological Risk Assessment of Organic Micropollutants in the Lower Reaches of the Yangtze River, China: A Case Study of Water Diversion." *Environ. Pollut.* **2018**, *239*, 223–232.

2. Isidori, M.; Lavorgna, M.; Nardelli, A.; Parrella, A.; Previtera, L.; Rubino, M. "Ecotoxicity of Naproxen and Its Phototransformation Products." *Sci. Total Environ.* **2005**, *348*, 93–101.

3. Backhaus, T.; Scholze, M.; Grimme, L. H.. "The Single Substance and Mixture Toxicity of Quinolones to the Bioluminescent Bacterium Vibrio Fischeri." *Aquat. Toxicol.* **2000**, *49*, 49–61.

4. Chen, Zhi-Feng; Guang-Guo Ying. "Occurrence, Fate and Ecological Risk of Five Typical Azole Fungicides as Therapeutic and Personal Care Products in the Environment: A Review." *Environ. Intern.* **2015**, *84*, 142–53.

5. Paredes, E.; Perez, S.; Rodil, R.; Quintana, J.B.; Beiras, R. "Ecotoxicological Evaluation of Four Uv Filters Using Marine Organisms from Different Trophic Levels Isochrysis Galbana, Mytilus Galloprovincialis, Paracentrotus Lividus, and Siriella Armata." *Chemosphere* **2014**, *104*, 44–50.

| 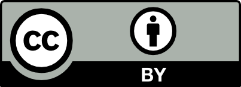 | © 2019 by the authors. Submitted for possible open access publication under the terms and conditions of the Creative Commons Attribution (CC BY) license (http://creativecommons.org/licenses/by/4.0/). |
| --- | --- |
